# Experiences of attending prenatal ultrasounds during the COVID‐19 pandemic in Australia: A cross‐sectional survey

**DOI:** 10.1111/birt.12867

**Published:** 2024-08-23

**Authors:** Helen J. Nightingale, Christina Watts, Kim Pham

**Affiliations:** ^1^ Rural Department of Nursing & Midwifery La Trobe University Bendigo Victoria Australia; ^2^ Faculty of Medicine and Health The University of Sydney Sydney Australia; ^3^ Department of Women's & Children's Services Bendigo Health Bendigo Australia

**Keywords:** pandemic, prenatal care, prenatal ultrasonography, psychological trauma

## Abstract

**Background:**

Prenatal ultrasounds form an important part of routine maternity care in Australia and indeed internationally. The COVID‐19 pandemic necessitated rapid changes in society and healthcare to curb transmission, with evidence demonstrating detrimental impacts on childbearing women associated with these restrictions. However, experiences with pandemic restrictions for prenatal ultrasounds in relation to distress, patient expectations, and satisfaction are largely unknown. This study aimed to explore the experiences of pregnant women attending prenatal ultrasound during the pandemic in Australia.

**Methods:**

A cross‐sectional online survey of people in Australia who had undergone at least one prenatal ultrasound during the period of maternity care restrictions was performed. The survey included validated tools for assessing post‐traumatic stress, satisfaction, and expectations with maternity care.

**Results:**

A total of 1280 responses were obtained. Almost 37% of respondents returned a PCL‐C score consistent with probable post‐traumatic stress disorder. Unexpected ultrasound findings or a high PCL‐C score were more likely to have higher expectations and lower levels of satisfaction with their maternity care experience. Having an ultrasound for pregnancy loss, fetal abnormality, and/or a prior post‐traumatic stress disorder diagnosis were the strongest factors correlating with a high PCL‐C score.

**Discussion:**

The prevalence of post‐traumatic stress symptoms in the study population is concerning and elucidates the distress experienced in association with prenatal ultrasounds during pandemic restrictions in Australia. Maternity services should acknowledge the high levels of service consumers with post‐trauma symptoms and consider trauma‐responsive maternity care adaptations in response to adverse perinatal outcomes for those afflicted with post‐trauma and distress‐related symptoms.

## Graphical abstract

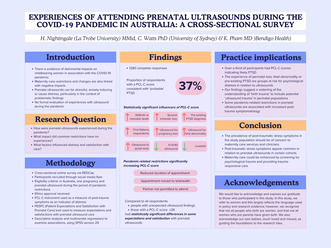

## 1 INTRODUCTION

Ultrasound examinations form part of routine maternity care, and Australian practice guidelines provide guidance on timing of ultrasound examinations in pregnancy. Prenatal ultrasounds provide an important opportunity to accurately date the pregnancy, confirm normal development, or conversely, identify possible problems and enable further care^1^; they also offer a chance for the woman and partner to connect with their developing baby, contributing to a positive pregnancy experience, providing reassurance, strengthening their relationship, and assisting the transition to parenthood.^2^, ^3^

Unexpected, concerning, or uncertain findings during a scan (such as twin pregnancy, fetal abnormality, or placental abnormality) can prompt feelings of shock, anxiety, or stress, result in reduced attachment to the baby, and may create distress in relation to subsequent ultrasound examinations, even if the findings are normal.^4^, ^5^ Rates of psychological distress or trauma are already acknowledged to be high in perinatal settings. This is increased in high‐risk cohorts, such as those experiencing complex pregnancies, perinatal loss, fetal anomaly, or complications such as hyperemesis gravidarum, where post‐traumatic stress disorder (PTSD) prevalence can be as high as 40%.^6^

Patient perspectives in healthcare are an important measure of quality of care received, and a conduit to service improvement.^7^ Fulfillment of expectations in maternity care is a consistent predictor of satisfaction with care, and both expectations and satisfaction with care are factors that provide a reflection of quality of care received.^8^, ^9^ Negative experiences, subjective distress, low support, and obstetric factors such as pregnancy complications increase the likelihood of subsequent mental health disorders, as well as perception of a traumatic event, post‐traumatic stress symptoms, and PTSD.^10^, ^11^, ^12^ The presence of these factors can have significant consequences for women and families, such as reduced parenting satisfaction, increased parenting stress, problematic attachment and bonding with the baby, infant development, family planning, and even reduced fertility.^13^, ^14^

During the COVID‐19 pandemic in Australia, rapid responses were implemented by governments and health services to suppress transmission of the virus.^15^ Maternity services were also impacted: masks became mandated; antenatal consults were reduced in frequency, duration, or moved to telehealth; birth education classes were ceased or moved to an online format; and there were limitations on in‐person supports for appointments—often preventing the partner or non‐pregnant parent from attending.^16^

Internationally, a wealth of evidence has examined experiences of maternity care during this time. The pandemic exacerbated feelings of psychological distress and mood disorders that are common in pregnancy,^16^, ^17^, ^18^ and a meta‐analysis highlighted rates of psychological distress around 70% in pregnant women during the COVID‐19 pandemic.^19^ In Australia and globally, maternity care restrictions intensified stress, anxiety, and distress for pregnant people^17^, ^20^ and some groups, such as people experiencing loss or pregnancy after loss, may have been disproportionately impacted by the restrictions.^21^ Partners of pregnant people felt isolated, distressed, and less connected to their babies as a result of the restrictions on their participation in maternity care.^22^

Existing evidence has determined that disallowing support people at prenatal ultrasounds had a negative impact on parental experiences and was distressing,^23^ and separation of the parents at a miscarriage diagnosis carried an associated emotional cost.^18^ However, formal evaluation of the impact of pandemic restrictions for prenatal ultrasounds on trauma and post‐traumatic stress symptoms and expectations and satisfaction with care is lacking.

This study aims to describe and understand the experience of pregnant women and people attending prenatal ultrasound during the COVID‐19 pandemic and examine the variables that influence participants' satisfaction with care and distress levels in association with their experiences.

## 2 METHODS

### 2.1 Procedure

A cross‐sectional online survey was administered through a secure platform (REDCap) with recruitment commencing in December 2022 and concluding in February 2023. An advertising flyer was created with a direct link to the open, online survey. This flyer was then distributed by means of social media and the researchers' networks. The flyer could be shared and thus snowball sampling was an additional recruitment strategy. All participants provided online informed consent before accessing the survey. Question order was not randomized. Only people who were in Australia, had experienced at least one pregnancy, and at least one ultrasound for pregnancy, during the period of pandemic restrictions (for the purpose of the study, considered from March 2020 to September 2022, when restrictions were still commonly in place in Australia), were eligible to participate. The strengthening the reporting of observational studies (STROBE) in Epidemiology guidelines for reporting on cross‐sectional studies and the Checklist for Reporting Results of Internet E‐Surveys (CHERRIES) were used to report the findings of this study.^24^

### 2.2 Ethical considerations

The study received approval from [redacted in line with journal requirements]. Participation was anonymous and participants were advised that they could stop participating at any point by simply exiting their browser. The survey preamble informed participants about expected time commitment for completing the survey, and the research team gave details about data storage and confidentiality. It acknowledged that the recall of their experiences may be distressing and contact details for the research team as well as a multitude of support and counseling services were provided. Participants were not offered incentives to participate.

### 2.3 Measures

Biographical data were collected by means of sociodemographic questions and questions about medical, psychological, and obstetric history. We evaluated the prevalence of post‐trauma symptoms as an indicator of distress experienced in relation to prenatal ultrasounds during the period of the pandemic by administering the PCL‐C instrument, which consists of 17 questions corresponding to the DSM‐5 (Diagnostic and Statistical Manual of Mental Disorders, 5th edition) symptom criteria for PTSD.^10^, ^25^ Participants respond with a Likert‐type scale (1 = not at all, 5 = extremely) to evaluate the level of distress experienced for each item in relation to their experience with prenatal ultrasounds during the period of the pandemic in Australia. A self‐administered PCL‐C questionnaire is validated for screening for PTSD,^10^ including in pregnant populations.^26^ The PCL‐C total score ranges from 17 to 85, and a score of ≥26 is considered indicative of PTSD in pregnant populations.^26^ In the present study, the PCL‐C demonstrated excellent internal consistency with a Cronbach's alpha of 0.96.

Additionally, we evaluated satisfaction and expectations in relation to prenatal ultrasound by means of 19 questions drawn from the Patient Expectations and Satisfaction with Prenatal Care (PESPC) tool, a validated instrument for measuring satisfaction with prenatal care.^27^ The final survey consisted of 67 questions. Where possible, wording for questions was amended by the research team who have personal experiences with perinatal or baby loss to ensure questions were appropriate and sensitive. The survey was piloted with the target population to ensure sensitivity and clarity of the wording of questions.

### 2.4 Analysis

Data were imported to IBM Statistical Package for Social Sciences (SPSS) version 29. Descriptive analysis was performed for demographic, medical, psychological, and obstetric data. Multivariate regressions were performed to examine associations between a high PCL‐C score and independent variables. All significance levels were set at *p* = 0.05.

## 3 RESULTS

Of a total of 1416 entries, 1280 participants progressed beyond initial demographic questions. Participant demographics are shown in Table 1. The majority were of white or European background, with more than half of respondents having a Bachelor's degree or higher.

**TABLE 1:** Participant demographics.

	*n* (%)
Age (*n* = 1280)	
18–30 years	387 (30.1)
31–40 years	877 (68.1)
41–45 years	22 (1.7%)
Older than 45	1 (0.1%)
Sex (*n* = 1279)	
Female	1271 (99.3)
Other	1 (0.1)
Declined to respond	7 (0.5%)
Region of birth (*n* = 1141)	
Oceania	995 (87.2)
Asia	48 (4.2)
Africa	15 (1.3)
Caribbean	2 (0.2)
Europe	59 (5.2)
North America	17 (1.5)
South America	4 (0.4)
Ethnicity (participants could select multiple options) (*n* = 1251)	
White European	1104 (88.6)
Indigenous/First Nations	21 (2.6)
Black African	1 (0.1)
Hispanic	8 (0.6)
Northern African	2 (0.2)
Middle Eastern	10 (0.8)
South Asian	6 (0.5)
South East Asian	24 (1.9)
East Asian	6 (0.5)
Māori	8 (0.6)
Pacific Islander	3 (0.2)
Mixed Race	39 (3.1)
Other	72 (5.8)
State/Territory (*n* = 1277)	
Australian Capital Territory	15 (1.2)
New South Wales	343 (26.9)
Northern Territory	9 (0.7)
Queensland	140 (11.0)
South Australia	50 (3.9)
Tasmania	23 (1.8)
Victoria	626 (49.0)
Western Australia	71 (5.6)
Highest level of education (*n* = 1287)	
Year 10 or equivalent	8 (0.6)
Higher school certificate/Year 12 or equivalent	84 (6.5)
Trade/apprenticeship	14 (1.1)
Certificate/diploma	218 (16.9)
Bachelor degree	525 (40.8)
Postgraduate degree/Master's degree or higher	231 (17.9)
Main occupation (*n* = 1280)	
Professional	717 (56.0)
Self‐employed	68 (5.3)
Stay‐at‐home parent	219 (17.1)
Manager/administrator	152 (11.9)
Tradesperson	9 (0.7)
Sales or Service worker	42 (3.3)
Laborer	2 (0.2)
Other	71 (5.5)
Employment status (*n* = 1283)	
Full‐time	334 (26.0)
Part‐time	579 (45.1)
Contract/temporary/casual	64 (5.0)
Unemployed	5 (0.4)
Unable to work	4 (0.3)
Stay‐at‐home parent	266 (20.7)
Other	29 (2.3)
Declined to respond	2 (0.2)
Relationship status (*n* = 1288)	
Single	6 (0.5)
Married/de facto	1246 (96.7)
In a relationship	31 (2.4)
Separated/divorced	4 (0.3)
Other	1 (0.1)
Annual household income, approximate ($AUD) (*n* = 1287)	
0–less than 40,000	14 (1.1)
40,000–less than 75,000	66 (5.1)
75,000–less than $130,000	451 (35.0)
130,000 or more	710 (55.2)
Declined to respond	46 (3.6)
Disability status (*n* = 1271)	
Considered or diagnosed as having a disability	25 (2.0)
No disability	1245 (98.0)
Declined to respond	1 (0.1)

### 3.1 Medical and obstetric characteristics

Participant medical and obstetric characteristics are presented in Table 2. Most participants experienced 1 or 2 pregnancies over the period of pandemic restrictions. Pre‐existing mental health conditions were common, with 40% of participants reporting a pre‐existing anxiety diagnosis, 25% reporting a pre‐existing depression diagnosis, and 7% identified an existing PTSD diagnosis. Most (64%) planned to birth in a public hospital, and 64% were engaged with a model of care affording at least some degree of continuity of care. The vast majority (91%) experienced a live birth during the period of pandemic restrictions, while almost 20% reported experiencing a first‐trimester miscarriage during this period. The mean PCL‐C score was 27.5, and almost 37% of respondents had a PCL‐C score of ≥26 indicating a high probability of PTSD.

**TABLE 2:** Participant medical and obstetric characteristics.

	*n* (%)
Number of pregnancies, total (*n* = 1251)	
1	419 (33.5)
2	421 (33.6)
3	241 (19.2)
4	104 (8.3)
5 or more	66 (5.3)
Number of pregnancies during pandemic restrictions period (March 2020 to end of 2022) (*n* = 1249)	
1	732 (58.6)
2	398 (31.9)
3	96 (7.7)
4	14 (1.1)
5 or more	9 (0.7)
Number of babies birthed, total (including babies born after 20 weeks of pregnancy, and any babies not still living)	
0	62 (5.0)
1	650 (51.9)
2	414 (33.1)
3	101 (8.1)
4	19 (1.5)
5 or more	6 (0.5)
Use of assisted reproduction technology (*n* = 1248)	
Yes	171 (13.7)
No	1076 (86.2)
Declined to respond	1 (0.1)
Current or prior mental health diagnosis (*n* = 1275)	
Anxiety	510 (40.0)
Depression	312 (24.5)
Bipolar disorder	9 (0.7)
Post‐traumatic stress disorder	90 (7.1)
Other	42 (3.3)
None of the above	691 (54.2)
Planned place of birth for pregnancy/s during the time of pandemic restrictions (*n* = 1251)	
Home	73 (5.8)
Public hospital	800 (63.9)
Private hospital	306 (24.5)
Birth center	39 (3.1)
I had more than one pregnancy at this time and different planned places for birth	24 (1.9)
Unsure	9 (0.7)
Lead carer for pregnancy/s during the time of pandemic restrictions (*n* = 1252)	
Private midwife	91 (7.3)
Private obstetrician	343 (27.4)
Midwifery group/caseload practice	284 (22.7)
Team midwifery	85 (6.8)
Public hospital—clinicians on shift	237 (18.9)
GP/obstetrician shared care	152 (12.1)
I had more than one pregnancy at this time and different lead care providers	50 (4.0)
Unsure	10 (0.8)
Pregnancy outcome/s experienced during the time of pandemic restrictions (multiple outcomes able to be selected) (*n* = 1251)	
Live birth	1136 (90.8)
First‐trimester miscarriage	246 (19.7)
Second‐trimester miscarriage	17 (1.4)
Ectopic pregnancy	11 (0.9)
Termination of pregnancy	31 (2.5)
Stillbirth or neonatal death	28 (2.2)
Premature birth	61 (4.9)
Currently pregnant at the time of participating	185 (14.8)
PCL‐C measures (*n* = 1043)	
PCL‐C score M (SD)	27.5 (14.7)
PCL‐C ≥ 26 n (%)	384 (36.8)
PCL‐C 25th percentile score	17
PCL‐C 75th percentile score	32

### 3.2 Expectations and satisfaction with prenatal ultrasounds experience

Table 3 presents the mean response scores for the PESPC instrument. For the purposes of analysis, we classified responses of ‘not at all’ and ‘a little bit’ as low expectation or satisfaction, responses of ‘quite a bit’ or ‘extremely’ as high expectation or satisfaction, with a response of ‘moderately’ representing average expectation or satisfaction. Thus, a mean score of <2 indicates lower levels of expectation or satisfaction, a score between 2 and <4 indicates moderate expectation or satisfaction, and a score of ≥4 represents a higher level of expectation or satisfaction. Mean scores for those with unexpected ultrasound findings and those with a PCL score ≥26 were compared with the mean scores for all respondents, with a *p* value of <0.05 indicating statistically significant differences in scores (highlighted in bold).

**TABLE 3:** Expectations and satisfaction responses.

PESPC	Mean, *p* *	Low expectation or satisfaction %	Moderate expectation or satisfaction %	High expectation or satisfaction %
I expected to have my ultrasound visit/s take a long time				
All	2.94	28.8	41.4	29.7
Those with unexpected US findings	2.94, *p* = 1.00	29.7	39.0	31.3
Those with PCL score ≥ 26	3.08, *p* = 0.11	23.8	44.4	31.9
I expected to see the same provider in the case of needing more than one ultrasound				
All	2.28	60.1	14.1	25.8
Those with unexpected US findings	2.34, *p* = 0.43	59.1	12.7	28.2
Those with PCL score ≥ 26	**2.76, *p* < 0.001**	43.8	16.9	39.4
I expected the provider to care how I felt mentally as well as physically				
All	3.84	14.9	17.3	67.9
Those with unexpected US findings	**3.99, *p* = 0.014**	11.6	15.8	72.6
Those with PCL score ≥ 26	**4.15, *p* = 0.012**	9.3	11.2	79.5
I expected the provider to be gentle during the examination				
All	4.43	3.2	5.7	91.1
Those with unexpected US findings	4.48, *p* = 0.22	2.3	8.3	91.7
Those with PCL score ≥ 26	4.48, *p* = 0.44	3.7	5.0	91.3
I expected to have my problems listened to				
All	4.00	10.8	15.5	73.7
Those with unexpected US findings	4.05, *p* = 0.50	10.2	14.3	75.4
Those with PCL score ≥ 26	4.14, *p* = 0.13	9.9	11.2	78.9
I am satisfied with the explanation the provider gave to me of what was going to happen				
All	3.74	14.9	20.7	64.4
Those with unexpected US findings	**3.56, *p* = 0.003**	21.4	21.4	57.1
Those with PCL‐score ≥ 26	**3.29, *p* < 0.001**	30.0	24.4	45.6
I am satisfied with the explanation the provider gave to me about how things were going following the ultrasound/s				
All	3.66	16.4	22.6	61.1
Those with unexpected US findings	**3.43, *p* < 0.001**	23.4	23.8	52.8
Those with PCL score ≥ 26	**3.25, *p* < 0.001**	19.3	24.2	46.6
I am satisfied with the way I was treated				
All	3.74	15.3	18.1	66.6
Those with unexpected US findings	**3.54, *p* = 0.001**	20.3	23.8	57.3
Those with PCL score ≥ 26	**3.12, *p* < 0.001**	35.4	18.6	46.0
I am satisfied with the quality of care I received				
All	3.77	14.1	18.3	67.6
Those with unexpected US findings	**3.58, *p* = 0.002**	18.5	22.7	58.8
Those with PCL score ≥ 26	**3.19, *p* < 0.001**	35.0	21.3	43.8
I am satisfied with not having to repeat my story every time I had an ultrasound				
All	2.96	39.9	21.3	38.8
Those with unexpected US findings	**2.63, *p* < 0.001**	51.9	19.3	28.8
Those with PCL score ≥ 26	**2.26, *p* < 0.001**	63.4	19.9	16.8
I am satisfied with being able to ask questions without embarrassment				
All	3.76	13.6	21.3	65.1
Those with unexpected US findings	**3.63, *p* = 0.02**	17.9	22.9	59.3
Those with PCL score ≥ 26	**3.40, *p* < 0.001**	22.4	29.8	47.9
I am satisfied with the way the staff expressed concern about my personal situation				
All	3.44	20.3	27.2	52.5
Those with unexpected US findings	**3.27, *p* < 0.001**	26.7	26.5	46.9
Those with PCL score ≥ 26	**2.99, *p* < 0.001**	36.9	26.9	36.3
I am satisfied with the way my medical/obstetric complications were managed				
All	3.59	18.2	22.2	59.6
Those with unexpected US findings	**3.37, *p* < 0.001**	27.2	20.6	52.1
Those with PCL score ≥ 26	**3.12, *p* < 0.001**	35.4	17.4	47.2
I am satisfied with the amount of time I waited to be seen for my ultrasound/s				
All	3.53	18.2	24.3	57.5
Those with unexpected US findings	**3.36, *p* = 0.005**	23.1	25.6	51.3
Those with PCL score ≥ 26	**3.18, *p* < 0.0002**	30.0	23.1	46.9
I am satisfied with the total amount of time I spent at the health service in relation to the ultrasound				
All	3.64	14.1	24.8	61.2
Those with unexpected US findings	3.49, *p* = 0.39	17.9	28.0	54.1
Those with PCL score ≥ 26	**3.25, *p* = 0.001**	24.5	30.2	45.2
I am satisfied with the examination room of the ultrasound/s				
All	3.98	8.1	15.7	76.2
Those with unexpected US findings	**3.87, *p* = 0.04**	10.3	18.2	71.6
Those with PCL score ≥ 26	**3.60, *p* < 0.001**	19.0	15.8	65.2
I am satisfied with my ability to schedule ultrasound visits at a time convenient for me				
All	3.22	30.8	22.0	47.1
Those with unexpected US findings	**3.00, *p* = 0.002**	36.4	22.8	40.8
Those with PCL score ≥ 26	**2.85, *p* < 0.001**	44.4	17.5	38.1
I am satisfied with how easy it was to reschedule my ultrasound visits				
All	3.07	34.9	23.8	41.4
Those with unexpected US findings	**2.87, *p* = 0.006**	41.1	22.9	36.0
Those with PCL score ≥ 26	**2.60, *p* < 0.001**	51.3	17.9	30.8
I am satisfied with the number of ultrasounds I had				
All	3.77	14.6	18.6	66.8
Those with unexpected US findings	**3.59, *p* = 0.005**	19.4	19.6	61.0
Those with PCL score ≥ 26	**3.31, *p* < 0.001**	26.1	24.2	49.6

*Note:* Italicised values indicate *p* values to distinguish from the mean values which are not italicised. Bold values indicate results that are statistically significant.

* Compared with all.

In the expectations scale, the highest average score was obtained for ‘provider gentleness’ with 91% of all responses indicating high expectations for this question; the lowest average score related to the expectation of seeing the same provider in the setting of needing more than one ultrasound (60% of all respondents indicating low expectations for this). Subgroup analysis of responses demonstrated that those with a high PCL‐C score had statistically higher expectations for seeing the same provider.

In the satisfaction scale, the highest score was obtained for satisfaction with the ultrasound examination room (76% of all respondents had high satisfaction), although those with unexpected ultrasound findings or high PCL‐C scores had significantly lower satisfaction in this factor. The lowest satisfaction score was obtained for ‘not having to repeat my story every time I had an ultrasound’, with almost 40% of participants indicating low satisfaction with this factor. Satisfaction scores were significantly lower for those with unexpected ultrasound findings (52%) and/or those with a high PCL‐C score (63%).

### 3.3 PCL score and distress measures

A total of 361 (35%) participants completing the PCL‐C tool returned a score of ≥26, considered sufficient for a probable PTSD diagnosis.^26^

Multivariate analysis using standard multiple regression was used to determine the significance of association between a positive PCL‐C score (≥26) and independent variables (Table 4). Results demonstrate that the independent variables explain a significant amount of the variance in PCL‐C scores (*R* ^2^ = 0.426, adjusted *R* ^2^ = 0.375 *p = <0.001*). Stillbirth or neonatal death, second‐trimester pregnancy loss, and pre‐existing PTSD diagnosis were the strongest predictors for a positive PCL‐C score. First Nations status, ultrasound for pregnancy loss, fetal abnormality, or as a guide for other investigations also significantly correlated with increased PCL‐C scores. Live birth and having a first‐trimester ultrasound between 11 and 14 weeks were the only significant protective factors correlating with a decrease in PCL‐C score. Of the pandemic restrictions for prenatal ultrasounds, prohibition of partner attendance, reduced appointment duration, and appointments moved to telehealth were all significantly correlated with an increase in PCL‐C score. Collinearity diagnostics suggested only weak correlations between independent variables; multicollinearity diagnostics were within acceptable limits.

**TABLE 4:** Significant predictors of PCL‐C score.

Factor	Unstandardized B	Standardized coefficients beta	t	p
Stillbirth/neonatal death	21.366	0.214	6.663	**<0.001**
Second‐trimester pregnancy loss	10.765	0.085	3.008	**0.003**
PTSD diagnosis	8.466	0.148	5.024	**<0.001**
First Nations	7.161	0.060	2.123	**0.034**
Ultrasound for pregnancy loss				
Ultrasound for fetal abnormality	3.863	0.151	3.287	**<0.001**
Ultrasound to guide for other tests	4.387	0.064	2.183	**0.029**
Live birth	−6.104	−0.120	−3.222	**0.001**
First‐trimester ultrasound 11–14 weeks	−3.421	−0.076	−2.497	**0.013**
Partner not permitted to attend	2.418	0.075	2.518	**0.012**
Ultrasound appointment reduced duration	3.957	0.101	3.512	**<0.001**
Ultrasound appointment moved to telehealth	2.580	0.058	2.083	**0.038**

*Note:* Bold values indicate results that are statistically significant.

## 4 DISCUSSION

To our knowledge, this is the first study to specifically examine pregnant women's and people's experiences of prenatal ultrasound during the pandemic in relation to expectations, satisfaction, and distress. These findings add to existing evidence that many pandemic‐responsive maternity care restrictions, such as reduced frequency or duration of ultrasounds and the exclusion of support person attendance, were correlated with increased distress and post‐traumatic stress symptoms.

A high proportion (37%) of respondents in this study returned a PCL‐C score consistent with probable PTSD, indicating high levels of distress and post‐trauma stress symptoms in relation to their experiences. This contrasts strongly with a previous meta‐analysis, indicating a PTSD prevalence of around 3% in general prenatal populations and 19% in high‐risk populations,^6^ although an American study exploring the impact of maternity care changes on birth trauma during the pandemic reported 72% of their sample were partially symptomatic for PTSD.^28^ Distress and trauma are significantly correlated with adverse perinatal outcomes,^29^ and may progress to diagnosable mental health conditions such as PTSD. Stress, distress in pregnancy, post‐trauma symptoms, and PTSD can all have significant consequences for mothers, babies, and families across the lifespan, including adverse effects on fetal brain development and infant neurodevelopment,^30^, ^31^ insecure or disorganized parent–baby attachment style,^32^, ^33^ reduced parenting satisfaction,^34^ and negative effects on lactation^35^ and even on future fertility and reproductive choices.^36^, ^37^ The serious and wide‐reaching consequences of distress, trauma, and PTSD occurring in the perinatal period need greater consideration in maternity care pathways, such as improved identification, responses, and treatment of the high proportions of pregnant people experiencing these conditions, particularly in marginalized or already disadvantaged populations, as our findings indicated higher PCL‐C scores in many of these groups.

Most participants in our study demonstrated moderate levels of expectations and satisfaction with elements of their prenatal ultrasound care, however, expectations and satisfaction levels were negatively affected if participants experienced unexpected ultrasound findings or returned a high PCL‐C score. While it is conceivable that these latter cohorts would have different care needs, the study results suggest that these needs were not met. The PESPC question relating to satisfaction with ‘not having to repeat your story at each ultrasound’ was rated lowest for satisfaction, and this was reflected more strongly in those with a PCL‐C score of 26 or more. While clinical care may require practitioners to confirm the clinical history or understanding of their patients, this finding demonstrates that requiring patients to re‐tell or repeat parts of their history is likely triggering or re‐traumatizing for people with trauma, and more sensitive considerations are needed. Increasing continuity of care for prenatal ultrasounds would not only address the above factor but also address the factor with lowest score relating to expectations (seeing the same provider for multiple ultrasounds).

Reduced satisfaction with perinatal care is linked to poorer outcomes,^9^ similarly, increasing satisfaction with maternity care services can result in better health outcomes for mothers and babies.^7^ Thus, it is possible that measures to address and increase satisfaction with prenatal care, particularly in at‐risk groups, may also improve outcomes. Existing guidelines provide evidence‐based guidance in the setting of unexpected or concerning prenatal ultrasound findings.^38^, ^39^ Information quality, provider empathy, privacy, and individualized language are factors valued by consumers in these circumstances^39^; and health services should prioritize practices, processes, and clinical supports for sonographers in order to facilitate parent‐centered care. These factors align with trauma‐informed practice principles,^40^ which strengthens the argument for integrating care for trauma‐related needs into maternity care and would address the additional needs of people with trauma‐ and stress‐related symptoms and disorders without harm to clinicians or other consumers. This approach can also assist in buffering the impact of any future public health measures or changes to care. Integrating trauma‐responsive principles in maternity care would require systematic education and training for maternity care clinicians with regards to psychological trauma and its sequelae, implementation of trauma‐sensitive screening and response strategies, and policies and procedures that seek to avoid re‐traumatization of individuals.^40^, ^41^

Pregnancy in 2020, representing the initial stages of the pandemic, was associated with a high PCL‐C score, but pregnancy in 2021 and 2022 was not. Other research suggests this likely reflects the panic that was experienced at the onset of the pandemic in relation to the unknown of the pandemic on perinatal outcomes and the rapid changes to maternity care, as well as public health measures to limit virus transmission.^16^ However, many of these changes and measures acted to reduce social support available to pregnant women,^42^ and our findings add to existing evidence that removal of partners or support for pregnant people during ultrasounds had negative consequences on parental experiences, maternal support, and bonding.^23^ Having support during the perinatal period is a crucial element of safe and respectful maternity care, providing emotional support and comfort, improving satisfaction with the experience of perinatal outcomes,^28^, ^43^ and the presence of a support person is strongly desired by pregnant women.^4^ Limitation of support denies pregnant people access to this essential element of pregnancy and the benefits associated with support. Distress and post‐traumatic stress symptoms are also experienced by partners of pregnant people after traumatic events.^44^ Partners who feel excluded during perinatal care are more likely to develop distress, with possible subsequent implications on their relationships, parent–child bonding, and child development.^10^, ^23^ The perspectives and experiences of partners were not examined as part of this study, however, their perceptions of maternity care during the pandemic are important considerations for future research.

Additionally, results showed that vulnerable groups may be disproportionately impacted, which correlates with other research indicating social, racial, and ethnic disparities in post‐traumatic symptomatology in childbearing women.^45^, ^46^ In particular, anxiety and depressive symptoms are predictive of low social support in pregnant women^46^; the restriction of accompanying support in prenatal care may have exacerbated mental health symptomatology or perception of poor support in many pregnant women. The inconsistency of ultrasound restrictions across health services also likely exacerbated existing inequalities and those who could access and afford private maternity care with fewer restrictions, such as those where partners were able to be present, did so.^18^

### 4.1 Implications for practice and policy

Notably, the findings of this study that relate to post‐trauma symptoms in association with prenatal ultrasound highlight the phenomenon of ‘ultrasound trauma’, affecting a considerable number of people accessing maternity care. Maternity care clinicians need to be aware of the potential for previous trauma in relation to prenatal ultrasounds, and, in alignment with trauma‐informed practice (TIP) principles, provide consumers with safe, individualized, and flexible choices for decision‐making and care relating to prenatal ultrasounds. Early and universal screening for traumatic stress conditions would allow maternity care clinicians to detect, support, and offer treatments and trauma‐responsive care options.^47^ However, most clinicians have little to no prior education and training relating to perinatal trauma and would require education to equip them with knowledge and skills to confidently implement trauma‐informed care.^48^

### 4.2 Strengths and limitations

This cross‐sectional study benefits from a large sample size which includes representation from many diverse groups of society. Importantly, this study contributes important knowledge to the available literature on perinatal psychological trauma and trauma symptomatology during pregnancy and in relation to prenatal ultrasounds.

Recall, response, and selection biases may impact our findings. Convenience sampling increases risk of selection bias by assessing a less representative sample of the study population,^49^ and our sample was highly educated and predominantly of white/European ethnicity. However, participant characteristics in this sample were in many ways reflective of the general childbearing population in Australia, but further research could examine other diverse populations such as First Nations, and culturally and linguistically diverse groups.

Given the effect of trauma on memory, it is plausible that participants in this study who experienced acute stress disorder or PTSD also remember their experiences in a different way (e.g., vivid, distorted, or repressed memory) than those unaffected by post‐trauma disorders. Similarly, time since the experience/s with prenatal ultrasound was not assessed, and this can affect recall and post‐trauma symptomatology. People with perceived positive or negative experiences with pregnancy ultrasounds during the pandemic may be more motivated to share their experience and participate; PTSD symptoms such as avoidance tendencies or intrusions may affect willingness to participate and/or disclose their experiences. Nonetheless, our sample comprised a higher proportion of individuals with a PTSD diagnosis, or a PCL‐C score consistent with probable PTSD, which is greater than the estimated prevalence of PTSD in pregnant or postpartum populations.^6^ This finding alone warrants serious consideration from maternity services, policymakers, and researchers with respect to provision of safe and trauma‐responsive care.

Participants needed to be able to read and write in English in order to complete the survey; the experiences of those without sufficient English language skills are not known. There are individual and organizational circumstances, such as practice variations, that influence experiences with prenatal ultrasounds and thus more research is required to determine the global generalizability of this study's findings. Our study comprised a high proportion of people with PTSD or a high PCL‐C score. The study design did not allow for gold‐standard clinical interviewing to confirm a PTSD diagnosis, however, while some contention exists suggesting that the PCL self‐report measure can inflate prevalence estimates of PTSD, a large systematic review and meta‐analysis demonstrated this measure produces results consistent with clinical interviewing and assessments for diagnosis and monitoring of post‐traumatic symptoms.^6^

## 5 CONCLUSIONS

This study provides a meaningful insight into the experiences of prenatal ultrasound during the pandemic in Australia. Of significant concern are the large numbers of participants returning PCL‐C scores consistent with PTSD, and the trauma and stress experienced in relation to pandemic ultrasound restrictions, including the limitation of support persons attending ultrasounds.

Prenatal ultrasounds where uncertain or abnormal findings were experienced, or involving pregnancy loss, were significantly associated with high PCL‐C scores, highlighting that ‘ultrasound trauma’ is a very common and valid phenomenon contributing to perinatal psychological trauma and stress‐related disorders.

The findings of this study represent an important opportunity to consider approaches to enhancing prenatal care in ways that would meet consumer expectations, optimize experiences, and reduce distress. Implementation of trauma‐informed education and practice across the spectrum of maternity care would result in service delivery that is truly person centered, safe, and compassionate, reduce the likelihood of situations that may prompt distress or a trauma response in traumatized individuals, and will not result in harm for clinicians or consumers.

## CONFLICT OF INTEREST STATEMENT

The authors declare no conflicts of interest.

## Data Availability

The data that support the findings of this study are available from the corresponding author upon reasonable request.
